# Pneumorrhachis as a complication of bronchial asthma: computed tomography findings

**DOI:** 10.1590/0100-3984.2016.0228

**Published:** 2018

**Authors:** Bruno Hochhegger, Klaus L. Irion, Daniela Hochhegger, Candice Simões Pires Santos, Edson Marchiori

**Affiliations:** 1 Grupo Hospitalar Conceição, Porto Alegre, RS, Brazil; 2 Manchester Royal Infirmary, Manchester, United Kingdom; 3 Universidade Federal do Rio de Janeiro (UFRJ), Rio de Janeiro, RJ, Brazil

**Figure 1:**
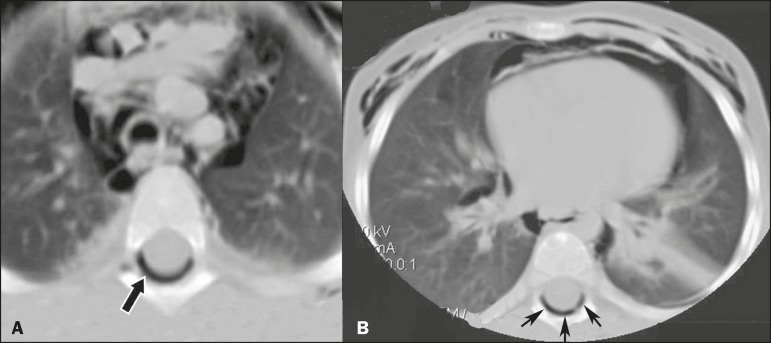
Chest CT scan, at the level of the upper (A) and lower (B) lobes, showing subcutaneous emphysema, pneumomediastinum, and pneumorrhachis (arrows).

Dear Editor,

We report the case of a 5-year-old male, previously diagnosed with bronchial asthma, who presented with a 3-day history of cough, fever, nausea, and vomiting. On physical examination, he was fully conscious and oriented but dyspneic. Body temperature was 38ºC. There was extensive subcutaneous bilateral emphysema involving the upper chest walls, neck, and axillary regions. Auscultation revealed extensive, bilateral expiratory polyphonic wheezes with few inspiratory crackles. Laboratory test results were normal. Low-dose computed tomography (CT) revealed pneumomediastinum, subcutaneous (cervical and thoracic) emphysema, and pneumorrhachis at the thoracic spine (Figure 1). The patient was treated with antibiotics, inhaled bronchodilators, systemic corticosteroids, high-flow oxygen, and other supportive measures. His six days in the hospital were uneventful, and he was asymptomatic at discharge.

Spontaneous pneumomediastinum is an uncommon entity, primarily affecting children and young adults. The presence of air within the spinal canal (pneumorrhachis) is a rare complication of spontaneous pneumomediastinum and is an exceptional imaging finding. Most commonly, pneumorrhachis is a consequence of trauma or is iatrogenic. It can be classified into two main groups: internal, or intradural, when gas is observed within the dural sac or subarachnoid space; and external, or extradural, when the air is within the spinal cord canal, external to the dural sac. External pneumorrhachis by itself is usually innocuous, whereas internal pneumorrhachis is often associated with major trauma and believed to be a marker of severe injury. Traumatic spinal leaks and penetrating spinal injuries are possible explanations for a direct route of intraspinal air entry. The air from the mediastinum can reach the spinal cord canal by dissecting the interstitium surrounding the vessels and nerves via the neural foramina or along the vascular and nerve root sheaths^(1-4)^.

Pneumorrhachis associated with bronchial asthma is extremely rare. In a recent review of the literature, no more than 13 cases describing pneumorrhachis caused by violent coughing due to bronchial asthma or acute bronchitis were identified. This form of pneumorrhachis is usually extradural. Pneumorrhachis in this context is typically asymptomatic and does not tend to migrate; it also reabsorbs spontaneously and completely, the air being passed directly into the blood within several days, without recurrence. Therefore, cases of pneumorrhachis are usually managed conservatively. Because of the rarity of the condition, there are no standard guidelines for the management of pneumorrhachis^(2)^.

The diagnostic tool of choice for pneumorrhachis is CT^(2,5)^. However, it can be difficult to differentiate between intradural and extradural pneumorrhachis on CT scans^(5,6)^. After the initial CT examination, the follow-up of patients with pneumorrhachis should primarily rely on clinical observations^(2)^. In the differential diagnosis, intraspinal gas collection, due to degenerative, malignant, inflammatory, and infectious diseases caused by gasforming organisms, has to be considered^(7-9)^. In conclusion, pneumorrhachis associated with asthma is usually self-limiting and without further therapeutic consequences.
